# Supplementary material for: Generation of squeezed vacuum state in the millihertz frequency band
Source: Light Sci Appl. 2024 Oct 17;13:294. doi: 10.1038/s41377-024-01606-y (PMC11487065; doi:10.1038/s41377-024-01606-y)
Supplement: Supplementary file 1 — Supplementary Information for: Generation of squeezed vacuum state in the millihertz frequency band [file 41377_2024_1606_MOESM1_ESM.docx]

**Supplementary Information for:**

**Generation of squeezed vacuum state in the millihertz frequency band**

**Li Gao^1,†^, Li-ang Zheng^1,†^, Bo Lu^1^, Shaoping Shi^1,*^, Long Tian^1,2^, and Yaohui Zheng^1,2,**^**

^1^State Key Laboratory of Quantum Optics and Quantum Optics Devices,

Institute of Opto-Electronics, Shanxi University, Taiyuan 030006, China.

^2^Collaborative Innovation Center of Extreme Optics, Shanxi University,

Taiyuan 030006, China.

^†^These authors contributed equally to this work.

[*ssp4208@sxu.edu.cn](mailto:*ssp4208@sxu.edu.cn)

[**yhzheng@sxu.edu.cn](mailto:**yhzheng@sxu.edu.cn)

This document provides supplementary material for “Generation of squeezed vacuum state in the millihertz frequency band”. It contains two parts: (1) Analysis of the optical mode matching efficiency, and (2) Temperature fluctuation of the periodically poled KTiOPO_4_ (PPKTP) crystal due to pump power noise.

**1. Analysis of the optical mode matching efficiency**

Assuming a Gaussian beam is injected into an optical cavity, the factor affecting the mode matching efficiency between the beam and optical cavity includes misaligned parameter *ξ* and mismatched parameter *ο*. The misaligned parameter $\xi=(\varepsilon-q_{\text{P}}\alpha)\omega_{\text{P}}$ is related to axis offset *ε*, angle offset *α*, and waist size of the Gaussian beam *ω*_P_. The mismatched parameter *ο* can be written as $o=[(q^{*}-q_{\text{P}})/(q^{*}+q_{\text{P}})]^{1/2}$, which is related to the *q* parameter defined as $q(z)=\text{i}\frac{\text{π}\omega^{2}}{\lambda}+z$, where *q*(*z*) is the *q* parameter of the resonance cavity, and *q*_P_(*z*) represents the *q* parameter of the incident Gaussian beam.

Considering the negligible higher-order spatial modes coupling, the expression of the fundamental mode TEM_00_ coupling coefficient is:

$$A_{00}=W_{x}W_{y}E_{x}E_{y}$$

Where

$$W_{\nu}=(1-o_{\nu}^{4})^{1/4}$$

$$E_{\nu}=\exp\left\{\frac{q_{\nu}}{q_{\text{P}\nu}}[\frac{(1+o_{\nu}^{2})\xi_{\nu}^{2}}{2}-\frac{\varepsilon_{\nu}^{2}}{\omega_{\text{P}\nu}^{2}}]\right\}$$

Since the *x* and *y* direction expressions are the same, *ν* is used to represent the amplitude distribution direction of the Gaussian beam (*x* or *y*). It can be seen from Eq. (2) that *W_ν_* is only related to mismatched parameter *ο_ν_*, meanwhile, *E_ν_* in Eq. (3) is related to both misaligned parameter *ξ_ν_* and mismatched parameter *ο_ν_*. Ultimately, the mode matching efficiency *η* between a Gaussian beam and an optical cavity with perfect impedance matching is given by:

$$\eta=P_{00}/P=\left|A_{00}\right|^{2}$$

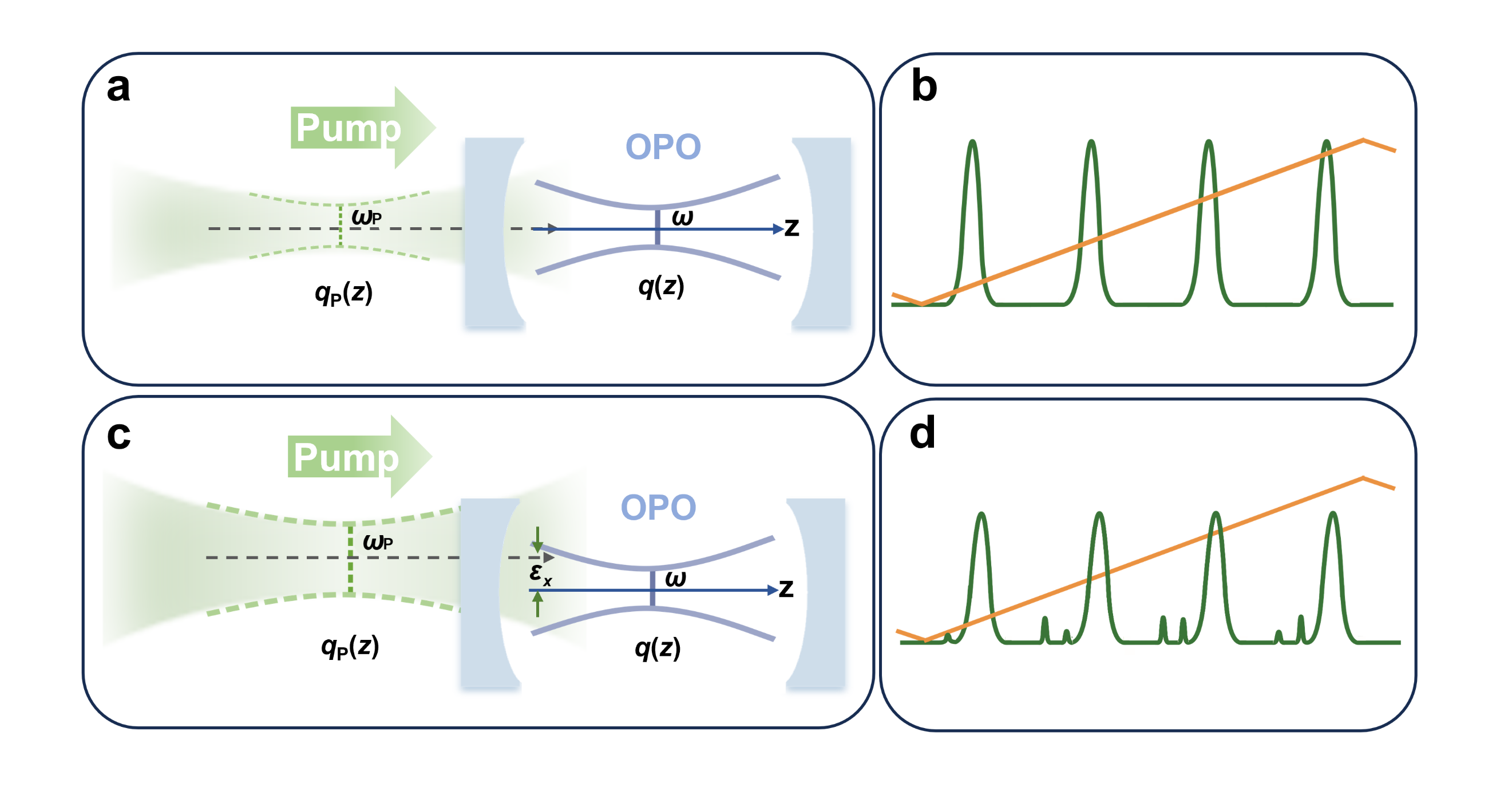


**Fig. S1 Schematic diagram of pump beam coupling with optical cavity.** **a** Perfect mode matching of the pump beam with the optical parametric oscillator (OPO). **b** Scanning OPO spectra of the pump beam. The green line represents the transmission curve of the pump beam through a scanned OPO cavity, meanwhile, the yellow line represents scanning voltage. **c** Imperfect mode matching of the pump beam with the OPO. The optical axis of the pump beam is offset by *ε_x_* against the resonator optical axis *z*. **d** Scanning OPO spectra of the pump beam under the condition of imperfect mode matching.

As shown in Fig. S1 **a** & **b**, we optimize the mode matching efficiency to the ideal initial status. Nonetheless, the displacement of the piezoelectric transducer (PZT) results in the pump beam displacement perpendicular to the beam axis, causing the mode matching efficiency between the pump beam and OPO to reduce, as shown in Fig. S1 **c & d**. The displacement between the actual optical axis and the ideal optical axis is *ε_x_* (considering *ε_y_* = 0), meanwhile, the tilt angle of the optical axis is *α_x_*=*α_y_*=0 and the waist size is *ω*_P_≠*ω*. We can express *q*_P_(*z*) and *q*(*z*) as the *q* parameter of the pump beam and OPO, respectively. Based on this the actual mode matching efficiency is given:

$$\eta=P_{00}/P=\left\{(1-o_{x}^{4})^{1/4}(1-o_{y}^{4})^{1/4}\exp\left\{\frac{q_{x}}{q_{\text{P}x}}[\frac{(1+o_{x}^{2})\xi_{x}^{2}}{2}-\frac{\varepsilon_{x}^{2}}{\omega_{\text{P}x}^{2}}]\right\}\right\}^{2}$$

In the experiment, the waist size of the pump beam (532 nm) is *ω*_P_*_ν_*=28 μm. For a common OPO cavity structure and PZT used for squeezed angle locking, the PZT-induced beam displacement *ε_x_* is about 2.8 μm. Similarly, the variation *z* of the optical path length is 2.8 μm. According to the Eq. (5), the mode matching efficiency is reduced from initial 100% to approximately 98.0% due to misaligned parameter *ξ* and mismatched parameter *ο*. The influence of the mismatch parameter *ξ* on the mode matching efficiency is negligible, when the effects of misaligned and mismatch errors are considered independently. We can see, from Fig. S2, that the mode matching efficiency is mainly limited by the displacement of the optical axis *ε_x_*.


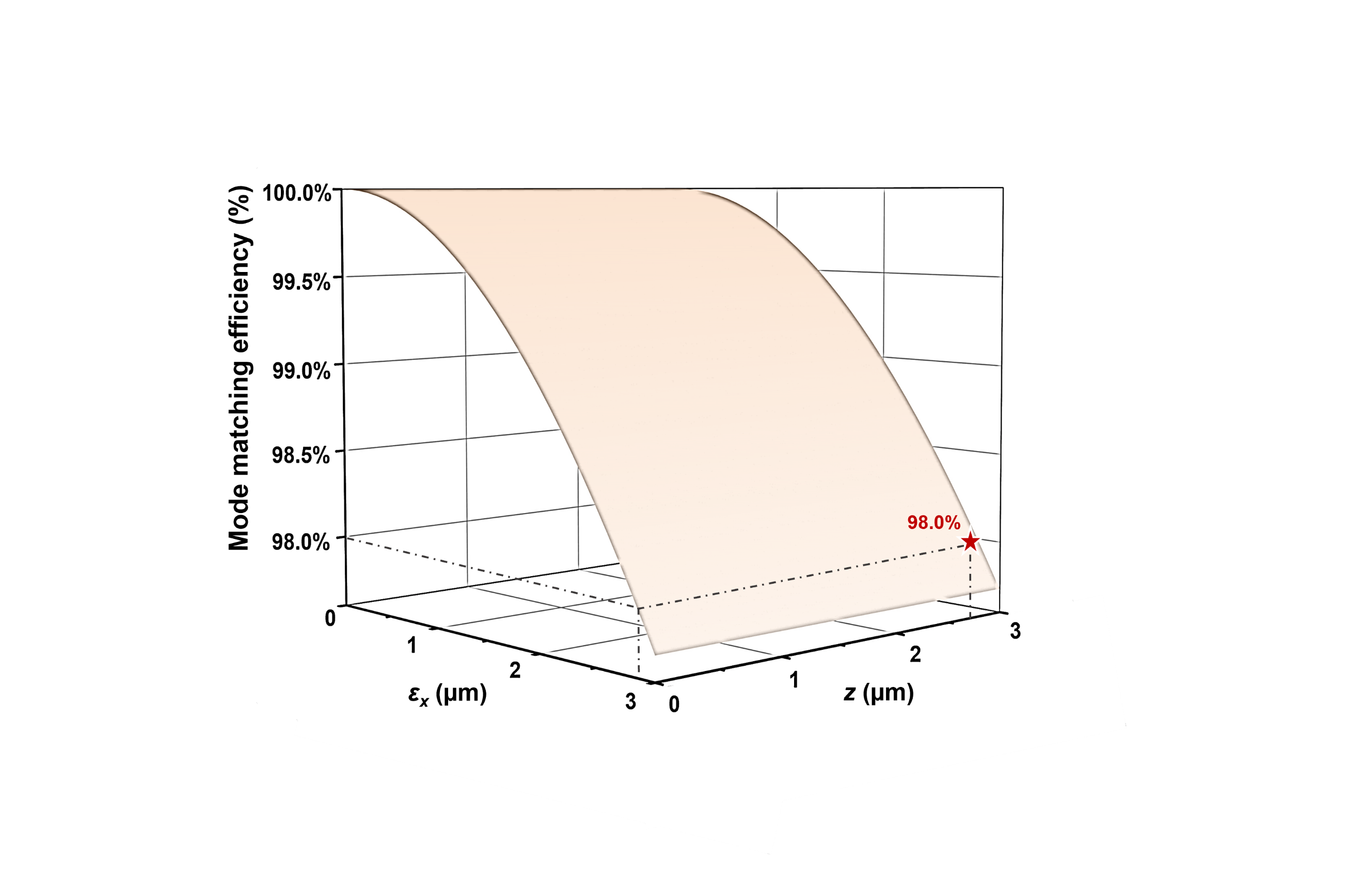


**Fig. S2 Mode matching efficiency as the function of the mismatched and misaligned errors.**

**2. Temperature fluctuation of the PPKTP crystal due to pump power noise.**

The temperature field distribution of the crystal is only affected by the intra-cavity circulating pump power when OPO operates in a steady state. Since the thermal conductivity of the crystal is greater than the heat exchange coefficient with the air, the end face of the PPKTP crystal satisfies the adiabatic condition. The crystal oven structure of the PPKTP crystal is shown in Fig. 1c of the main text, where the crystal is central heating by the absorbed pump laser and boundary cooling by a copper oven. There exists a thermal gradient across the cross-section of the PPKTP crystal. Although the boundary temperature is precisely stabilized, the central temperature inevitably fluctuates with the circulation power. The heat conduction within the crystal obeys Poisson's equation:

$$u_{xx}+u_{yy}=-\frac{h}{\chi}$$

The analytical solution of the above equation is:

$$u(x,y)=\sum_{n=1}^{\infty} \sum_{m=1}^{\infty} B_{nm}\sin\frac{n\text{π}}{a}x\sin\frac{m\text{π}}{b}y$$

The coefficient *B_nm_* in the Eq. (7) is:

$$B_{nm}=\frac{4P\beta ab}{\chi\text{π}^{2}(b^{2}n^{2}+a^{2}m^{2})}\times\int_{0}^{b} \int_{0}^{a} \exp\left[-2\frac{\left(x-a/2\right)^{2}+\left(y-b/2\right)^{2}}{\omega^{2}}\right]\times\sin\frac{n\text{π}}{a}x\sin\frac{m\text{π}}{b}ydxdy$$

where *h* is the thermal power density of the crystal, *χ* is the thermal conductivity of the crystal, and *β* is the laser absorption rate of the crystal. Besides, *a* and *b* are the length and width of the end face about the crystal respectively, and *ω* is the waist size of the pump beam. The normalized pump power in Eq. (8) is considered as $P=P_{0}/2\text{π}\int_{0}^{\infty} \exp(-2r^{2}/\omega^{2})rdr$ since the pump field in the OPO has an ideal Gaussian distribution, in which the intra-cavity circulating pump power *P*_0_ is related to the incident pump power *P*_in_.

The parameters taken here are *χ*=0.0033 W (mm*K)^-1^, *β*=0.004 mm^-1^, *a*=2 mm, *b*=1 mm, *ω*=28 µm and *P*_in_=7 mW. According to these theoretical parameters, we simulate the crystal temperature distribution caused by the experimental photothermal effect. The results are shown in Fig. S3. From Fig. S3, we can observe an obvious temperature gradient from the central to the boundary. The circulating power fluctuation coming from the change of the mode matching efficiency of 2% can induce temperature fluctuation in the order of 10^-3^ K at the center of the crystal, which is enough to break the initial optimal co-resonance status of OPO.


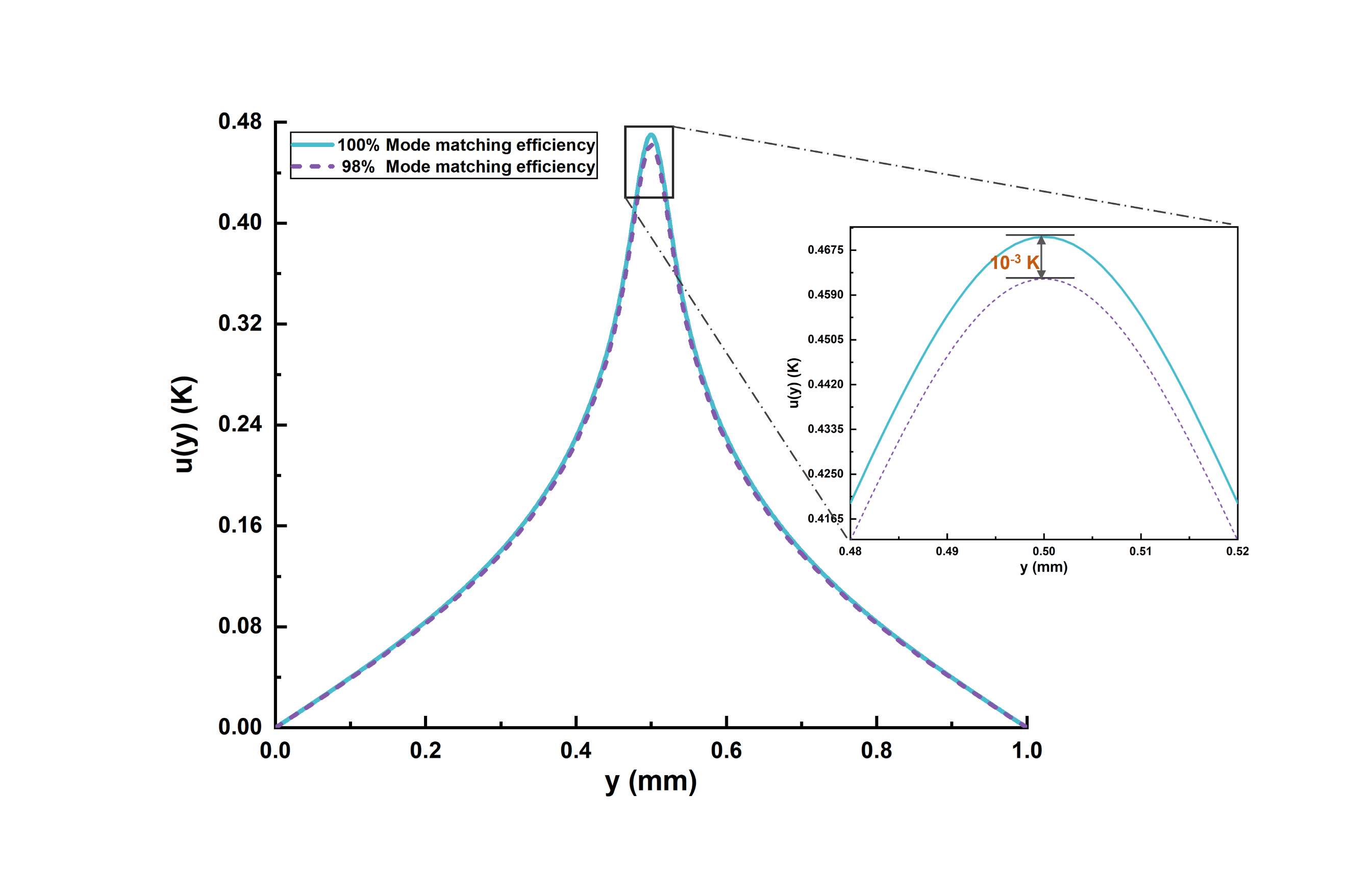


**Fig. S3 Temperature field distribution of PPKTP crystal from the central to the boundary.** Let *x* be fixed in the center of the pump beam, and *y* is a variable. The blue and purple lines represent the crystal temperature distribution corresponding to the 100% and 98% mode matching efficiency, respectively.

**Reference**

1. Sayeh, M. R., Bilger, H. R. & Habib, T. Optical resonator with an external source: excitation of the Hermite-Gaussian modes. *Appl. Opt.* **24**, 3756-3761 (1985).
2. Zhou, B. K. et al. in Laser Principle ,Vol. 7 (Beijing: National Defense Industry Press) Ch. 2 (2014).
3. Song, S. M., Hu, C. H. & Yan, C. X. Optical axis maladjustment sensitivity in a triangular ring resonator. *Appl. Opt.* **58**, 29-36 (2019).
4. Ling, L. et al. Temperature field of quadrate frequency crystal KTP in all-solid-state laser. *Laser Technology* **29**, 350-353 (2005).
5. Bierlein, J. D. & Vanherzeele, H. Potassium titanyl phosphate: properties and new applications. *J. Opt. Soc. Am. B* **6**, 622-633 (1989).
6. Nikogosyan, D. N. Nonlinear Optical Crystals:A Complete Survey. Ch. 2 (2015).
7. Schiller, S., Schneider, K. & Mlynek, J. Theory of an optical parametric oscillator with resonant pump and signal. *J. Opt. Soc. Am. B* **16**, 1512-1524 (1999).
8. Wang, Q. W. et al. Realizing a high-efficiency 426nm laser with PPKTP by reducing mode- mismatch caused by the thermal effect. *Opt. Express* **27**, 28534-28543 (2019).
